# Synergistic Effect of the* MTHFR* C677T and* EPHX2* G860A Polymorphism on the Increased Risk of Ischemic Stroke in Chinese Type 2 Diabetic Patients

**DOI:** 10.1155/2017/6216205

**Published:** 2017-03-20

**Authors:** Liang Ma, Yongwei Jiang, Xiaomu Kong, Meihua Yan, Tingting Zhao, Hailing Zhao, Qian Liu, Haojun Zhang, Yongtong Cao, Ping Li

**Affiliations:** ^1^Graduate School of Peking Union Medical College, Chinese Academy of Medical Sciences and Peking Union Medical College, Beijing, China; ^2^Clinical Laboratory, China-Japan Friendship Hospital, Beijing, China; ^3^Beijing Key Lab Immune-Mediated Inflammatory Diseases, Institute of Clinical Medical Science, China-Japan Friendship Hospital, Beijing, China; ^4^Department of Endocrinology, China-Japan Friendship Hospital, Beijing, China

## Abstract

The aim of this study was to investigate the relationship between the combined effect of* MTHFR* C677T (rs1801133) and* EPHX2 *G860A (rs751141) polymorphism and ischemic stroke in Chinese T2DM patients. This case-control study included a total of 626 Chinese T2DM patients (236 T2DM patients with ischemic stroke and 390 T2DM patients without ischemic stroke). The rs1801133 and rs751141 were genotyped using real-time polymerase chain reaction. Statistical analysis was performed with SPSS 17.0. Results showed that the combined effect of* MTHFR* TT and* EPHX2* GG or GA + AA genotype has a higher risk of ischemic stroke compared with the control group (combined effect of* MTHFR* CC and* EPHX2* GA + AA genotypes; OR = 3.46 and OR = 3.42, resp.; *P* = .001 and *P* = .002, resp.). The A allele showed marked association with a lower risk of ischemic stroke in patients with the lowest Hcy levels under additive, recessive, and dominant genetic models (OR = 0.45, OR = 0.11, and OR = 0.44, resp.; *P* = .002, *P* = .035, and *P* = .008, resp.), which was not observed in medium or high Hcy level groups. In conclusion, the T allele of rs1801133 and the G allele of rs751141 may be risk factors of ischemic stroke in the Chinese T2DM population.

## 1. Introduction

Type 2 diabetes mellitus (T2DM) is becoming increasingly prevalent throughout the world. In 2015, there were an estimated 415 million cases of DM worldwide, and that number is expected to increase to 642 million cases in 2040 according to the 7th Edition of the International Diabetes Federation (IDF)* Diabetes Atlas* [1]. In 2010, China alone had more than 113.9 million adults with diabetes and another 493.4 million with prediabetes [2]. Ischemic stroke is a major vascular complication of T2DM resulting in significant morbidity and mortality [3]. The risk of stroke in patients with T2DM is more than three times that of the general population [4]. Furthermore, genetics may play an important role, as East Asian patients with T2DM have a higher risk of developing strokes [5], and monozygotic twins have a higher concordance rate of stroke than dizygotic twins [6]. More studies are needed to explain these genetic differences, and effective strategies are required to facilitate earlier identification and prevention of ischemic stroke in persons with DM, particularly in China.

A high plasma homocysteine (Hcy) level is an independent risk factor for ischemic stroke [7]. Methylenetetrahydrofolate reductase (MTHFR) plays an important role in homocysteine metabolism.* MTHFR* C677T polymorphism (rs1801133) results in the substitution of alanine with valine (A222V), which causes reduced MTHFR activity and an elevated homocysteine level. Individuals with the* MTHFR* TT genotype have higher plasma homocysteine levels than those with CT or CC genotype [8]. Compared with the 677CC genotype, the homozygous 677TT and heterozygous 677CT genotypes have approximately 30% and 65%, respectively, of the enzyme activity of MTHFR [9]. Studies on the association between* MTHFR* C677T and ischemic stroke have shown conflicting results, ranging from no effect [10, 11] to a mild-to-moderate effect [12]. Results of the few investigations on the association between* MTHFR* C677T polymorphism and ischemic stroke in the Chinese T2DM population have also been uncertain [13, 14].

*EPHX2*, a gene that we studied, encodes soluble epoxide hydrolase (sEH), which degrades epoxyeicosatrienoic acids (EETs) to corresponding diols [15]. EETs are involved in regulation of long-term arterial blood pressure. Moreover, development of stroke also seems linked to the levels of EETs in the cerebral microvascular system [16]. However, reducing sEH activity by a small molecular inhibitor can increase EET levels and decrease their degradation [17]. One of the known* EPHX2 *SNPs is G860A polymorphism (rs751141), which results in a lower sEH activity [18]. The G860A polymorphism in the* EPHX2* gene has been found to be associated with the risk of ischemic stroke, but results were inconsistent among different studies [16, 19–23]. Another study found that the A allele of G860A is associated with a significantly lower risk of ischemic stroke in a Chinese population [23]; however, G860A was not found to be associated with an altered risk of ischemic stroke in a cohort of Americans of African or Caucasian descent [20]. To date, no study has investigated the association between* EPHX2 *G860A polymorphism and ischemic stroke in the Chinese T2DM population.

Both Hcy level and EETs are related to ischemic stroke disease, and* MTHFR* C677T polymorphism can elevate Hcy level and high Hcy level can upregulate sEH protein expression in vitro and in vivo [24]. Lone genetic abnormalities are rarely the exclusive cause of ischemic stroke. Based on these observations, we performed genetic association analyses in a cohort of Chinese T2DM population from Beijing, China, aiming to evaluate the association of the combined effect of* MTHFR* C677T and* EPHX2 *G860A on ischemic stroke.

## 2. Materials and Methods

This study was approved by the institutional ethics committee of the China-Japan Friendship Hospital, Beijing, China. Signed informed consent was obtained from all participants. A total of 626 persons with confirmed diagnoses of T2DM were included in this study. They were all hospitalized patients at the China-Japan Friendship Hospital between February 2015 and June 2016. This was a clinic-based case-control study. Among these participants, 236 individuals had a history of ischemic stroke and were defined as cases. The remaining 390 participants, who were diagnosed with T2DM for at least 10 years and had no history of ischemic stroke, were defined as controls, regardless of age and sex. (Controls were unlikely to have ischemic stroke in the future, which increased the sensitivity in detecting the associations.)

In addition, T2DM was diagnosed by the World Health Organization (WHO) 1999 criteria [25]. Ischemic stroke was diagnosed as an acute focal or global neurologic deficit lasting more than 24 hours without apparent cause other than that of vascular origin, consecutively confirmed by brain computed tomography (CT) or magnetic resonance imaging (MRI) scan within 72 hours from onset of the symptoms. Patients with cerebral hemorrhage, cerebral venous thrombosis, and brain tumor were excluded from the present study.

### 2.1. Data Collection

Demographic information, smoking habit, history of hypertension, body mass index (BMI), systolic blood pressure (SBP), diastolic blood pressure (DBP), hemoglobin A1C (A1C), high density lipoprotein cholesterol (HDL-C), low density lipoprotein cholesterol (LDL-C), total cholesterol (TC), triglycerides (TG), and homocysteine (Hcy) of each participant were obtained. Body weight and height were measured using standard methods and BMI was calculated as weight (kg) divided by height squared (m^2^). Resting blood pressure was measured twice according to standard protocol and the results were averaged. Serum concentrations of fasting TG, TC, LDL-C, HDL-C, and Hcy were measured using an automated biochemical analyzer (AU5800 Clinical Chemistry System, Beckman Coulter, Brea, CA, USA). A1C was measured using the D-10 Hemoglobin Testing System (Bio-Rad, Hercules, CA, USA).

### 2.2. DNA Extraction

DNA was extracted from peripheral blood using the QIAamp DNA Blood Mini Kit (Qiagen, Hilden, Germany) following the manufacturer's recommendations and then stored at −20°C or amplified immediately. Concentration of DNA was determined using the NanoDrop 1000 spectrophotometer (ThermoScientific, Waltham, MA, USA).

### 2.3. Amplification and Detection of* EPHX2* Gene R287Q Polymorphism

Genotyping was confirmed using TaqMan SNP Genotyping Assay (Applied Biosystems, Waltham, MA, USA). In all, 50 ng DNA was amplified in a 25 *μ*L reaction mixture containing 12.5 *μ*L of Premix Ex Taq (Probe qPCR) (Takara, Japan), 5 pmol of each primer (Applied Biosystems), and 3 pmol of each probe (Applied Biosystems) for the amplification of* EPHX2*. The primer and probe sequences were custom designed and synthesized by Applied Biosystems. The* EPHX2 *primer sequences were as follows: F: 5′-CGGGAGGAGCAGATGACTCT-3′ and R: 5′-TGGAGTGTGCCTGTTTGTTTTC-3′. The probe sequences were as follows: FAM-5′-CATAGCTAGGACC**C**GGTAACCTGCCT-3′-TAMRA and 5′VIC-5′-CCATAGCTAGGACC**T**GGTAACCTGCCT-3′-TAMRA. The* MTHFR *primer sequences were as follows: F: 5′-GGCTGACCTGAAGCACTTGAA-3′ and R: 5′-AGAAAAGCTGCGTGATGATGAA-3′. The probe sequences were as follows: FAM-5′-TCTGCGGGAGTCG-3′-MGB and VIC-5′-CTGCGGGAGCCGA-3′-MGB.

Amplification was performed using a real-time polymerase chain reaction (PCR) detector (LightCycler480, Roche Diagnostics, Penzberg, Germany) with a PCR temperature profile consisting of denaturation at 95°C for 10 minutes followed by 40 cycles of denaturation at 95°C for 15 seconds and then by annealing and elongation at 65°C for 60 seconds.

### 2.4. Genotyping Using DNA Sequencing

To confirm the genotyping results, 50 samples were randomly selected for DNA sequencing.* MTHFR* C677T polymorphism was amplified for DNA sequencing using the following custom designed primers: F: 5′-GTCTCTTCATCCCTCGCCTT-3′ and R: 5′-GAACTCAGCGAACTCAGCAC-3′.* EPHX2* G860A polymorphism was amplified for DNA sequencing using the following custom designed primers: F: 5′-TTACAGGAAGAAGGGGATGG-3′ and R: 5′-GGCAGGTAGAAGGCAAGACC-3′. Amplifications were performed using standard protocol and the PCR products were purified using the QIAquick PCR Purification Kit (Qiagen) and subsequently analyzed by direct sequencing with an automated DNA sequencer (3500 Genetic Analyzer, Applied Biosystems).

### 2.5. Statistical Analyses

Quantitative clinical data (age, BMI, blood pressure, duration of diabetes, A1C, TC, HDL-C, LDL-C, TG, and Hcy) were non-Gaussian distribution and presented as median (interquartile range), and Wilcoxon signed rank test was used to compare the differences in clinical characteristics between the ischemic stroke and control groups. Genotype distribution and allelic frequency were analyzed using the chi-square test. Deviations from Hardy-Weinberg were also tested using the chi-square test. Finally, multivariate logistic regression analyses were carried out to examine the association between* MTHFR* C677T and* EPHX2 *G860A polymorphism and risk of ischemic stroke adjusted for age, sex, BMI, history of hypertension, TC, TG, and HDL-C in the additive, recessive, or dominant models. To define these models, take SNP rs751141 as an example where A is the minor allele. For the dominant model, AA and GA were coded as 1 in the regression model and GG was coded 0. For the recessive model, AA was coded as 1 while GG and GA were coded as 0. For the additive model, AA, GA, and GG were coded as 2, 1, and 0, respectively. For SNP rs1801133, T is the minor allele.

Power calculation was performed by Quanto software (version 1.2.4, University of Southern California, Los Angeles, CA, USA). Data were analyzed with SPSS software (version 17.0, IBM, Armonk, NY, USA).* P *values of <.05 were considered significant.

## 3. Results

### 3.1. Baseline Characteristics

A total of 626 T2DM participants were included in this study. Cases included 236 patients with a history of ischemic stroke (127 males and 109 females) and controls included 390 patients without a history of ischemic stroke (241 males and 149 females) (Table 1). Variables, such as age, systolic blood pressure (SBP), and history of hypertension, were found to be elevated in ischemic stroke patients compared to controls (Table 1).

### 3.2. Distribution of* MTHFR* C677T and* EPHX2 *G860A Polymorphism

Distribution of allele frequencies of* MTHFR* C677T and* EPHX2 *G860A was in accordance with the Hardy-Weinberg equilibrium in both ischemic stroke and control groups.

Genetic distribution of* MTHFR *C677T (CC, CT, and TT) between T2DM participants with and without ischemic stroke (*P* = .020) as well as allele frequencies (C and T alleles) (*P* = .005) is shown in Table 2. Genetic distribution of* EPHX2* G860A (GG, GA, and AA) trended toward statistical difference between T2DM participants with and without ischemic stroke (*P* = .053) and allele frequencies (A and G alleles) were significantly different (*P* = .023) (Table 2).

### 3.3. Association of* MTHFR* C677T and* EPHX2 *G860A Polymorphism with Ischemic Stroke Risk

Results of the risk of ischemic stroke with* MTHFR* C677T and* EPHX2* G860A in these models were tabulated (Table 3).

In terms of the* MTHFR* C677T genotype, the additive, dominant, and recessive models were significantly different in participants with ischemic stroke compared to the control group (*P* = .005, *P* = .033, and *P* = .012, resp.). TT genotype was found to significantly increase the risk of ischemic stroke compared with the CC genotype (*P* = .006). These data suggest an increased risk effect of the T allele of* MTHFR* C677T polymorphism on ischemic stroke. After adjusting for age, sex, BMI, history of hypertension, TC, TG, and HDL-C, results were similar.

In terms of the* EPHX2* G860A genotype, the additive and dominant models were markedly different in participants with ischemic stroke than in the control group (*P* = .02 and *P* = .016, resp.). GA genotyping significantly decreased the risk of ischemic stroke compared with GG genotyping (*P* = .025). These data suggest a protective effect of the A allele of G860A polymorphism against ischemic stroke. The CI of the recessive model was wide owing to the small number of AA genotype participants and not found to significantly decrease the risk of ischemic stroke compared with GG genotyping (*P* = .182). After adjusting for age, sex, BMI, history of hypertension, TC, TG, and HDL-C, results were similar.

### 3.4. Association of the Combined Effect of* MTHFR* C677T and* EPHX2* G860A Polymorphism with Ischemic Stroke Risk

**Figure 1:**
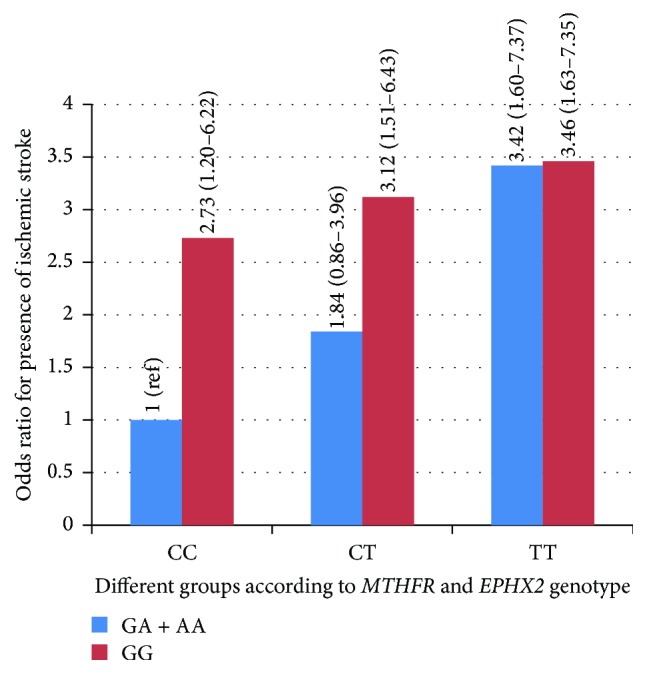
Risk of the combined effect of* MTHFR* C677T and* EPHX2* G860A polymorphism on ischemic stroke. Odds ratios (95% CI) are shown.

The combined effect of the* MTHFR* TT genotype and the* EPHX2* GG or GA + AA genotype was found to have a higher risk of ischemic stroke compared with the control group (combined effect of* MTHFR* CC genotype and* EPHX2* GA + AA genotype: OR = 3.46 and OR = 3.42, resp.; *P* = .001 and *P* = .002, resp.). When* MTHFR* polymorphism was CT genotype, the* EPHX2* GG genotype group had a higher risk of ischemic stroke compared with the control group (OR = 3.12; *P* = .002), whereas* EPHX2* GA + AA genotype group did not (OR = 1.84; *P* = .114). The combined effect of* MTHFR* CC and* EPHX2* GG genotypes also had a higher risk of ischemic stroke compared with the control group (OR = 2.73; *P* = .017) (Figure 1). After adjusting for age, sex, BMI, history of hypertension, TC, TG, and HDL-C, results were similar (Table 4).

### 3.5. *EPHX2 *G860A Polymorphism and Hcy Level Interaction

Participants were randomly divided into three equal groups based on Hcy levels (low: 4.29–10.7 *μ*mol/L; medium: 10.74–13.71 *μ*mol/L; high: 13.73–53.99 *μ*mol/L). The three genetic models that are additive, recessive, and dominant showed significant association with ischemic stroke in the low level Hcy groups in the unadjusted models (*P* = .002, *P* = .035, and *P* = .008, resp.), indicating that the A allele carriers in the low Hcy groups were at lower risk of ischemic stroke, while no significant association was observed in the medium (*P* = .579, *P* = .124, and *P* = .931, resp.) and high Hcy groups (*P* = .152, *P* = .879, and *P* = .102, resp.) in the adjusted models. After multivariate adjustment, we achieved similar results in the two models (Table 5).

## 4. Discussion

Stroke is the leading cause of death in China and second leading cause of death in the world [26]. To our knowledge, this study is the first to describe the association of the combined effect of* MTHFR* C677T and* EPHX2 *G860A polymorphism with ischemic stroke in T2DM patients. Genetic distribution of* MTHFR *C677T suggests that the presence of the T allele of C677T and G allele of G860A may be associated with a risk factor for ischemic stroke. In our study, the combined effect of the* MTHFR* TT and* EPHX2* GG or GA + AA genotypes resulted in a higher risk of ischemic stroke. When* MTHFR* polymorphism was CT genotype, the* EPHX2* GG genotype group had a higher risk of ischemic stroke, whereas the* EPHX2* GA + AA genotype group did not. Significant association of rs751141 and low Hcy level with the risk of ischemic stroke was observed, indicating that the A allele showed marked association with lower risk of ischemic stroke in all three genetic models. Based on our results, it can be concluded that the T allele of* MTHFR* C677T and G allele of* EPHX2* G860A polymorphism appear to impart susceptibility to ischemic stroke in the Chinese T2DM population.

Studies have demonstrated that hyperhomocysteinemia is an independent risk factor for stroke [7] and that Hcy is associated with* MTHFR* C677T polymorphism [27], which was validated in this current study (Supplementary Table  1, in Supplementary Material available online at https://doi.org/10.1155/2017/6216205). Furthermore, the frequency of the* MTHFR* 677TT genotype, which shows marked ethnic variation, is more common in China than in most European countries [28], with Chinese population having higher Hcy levels [29]. In our study, additive and recessive models of* MTHFR* C677T polymorphism were significantly different in T2DM participants with and without ischemic stroke. TT genotype was found to significantly increase the risk of ischemic stroke compared with CC genotype in unadjusted and adjusted models, which is consistent with the results in Chinese T2DM patients [14]. These data suggest an increased risk effect of the* MTHFR* C677T polymorphism on ischemic stroke in Chinese T2DM population.

The A allele of G860A has been found to exhibit markedly lower sEH metabolic activity of EETs in vitro [18]. Results of some studies have found a protective effect of decreased sEH activity against ischemic stroke [16, 19, 20, 23], whereas one study showed an association of the G860A allele with an increased risk of ischemic stroke [21]. Yet another investigation, which included 12 times more individuals than all previous studies combined, found no association between genetically reduced sEH activity and risk of ischemic stroke [22]. Our results showed that the additive and dominant models were significantly different in participants with ischemic stroke than in the control group, which is consistent with previous results [19, 20, 23]. There might be several mechanisms of the protective effect of the A allele of G860A against ischemic stroke. Studies in rodent models have shown that* EPHX2* deletion or pharmacologic inhibition of sEH activity could reduce experimental focal ischemic stroke [16, 30], which was at least partly due to increased levels of EETs. Moreover, in type 2 diabetic mice, inhibition of sEH was observed to improve glycemic status, postischemic reperfusion in the ischemic region, and stroke outcomes [31]. Additionally, an in vitro study demonstrated that the A allele of* EPHX2* G860A could protect neuronal cells from oxygen-glucose deprivation- (OGD-) induced cell death [32]. Since A allele carriers of rs751141 have a reduced level of sEH activity [33], we speculate that the protective effect of this allele against ischemic stroke as observed in the present study may be due to reduced sEH activity and the accumulation of EETs. The mechanism of this effect warrants further investigation.

It is well acknowledged that single genetic abnormalities are rarely the only cause of stroke. Thus, an interesting result of our study is that the combined effect of the* MTHFR* TT and* EPHX2* GG or GA + AA genotypes imparted a higher risk of ischemic stroke compared with the control group. When* MTHFR* polymorphism was CT genotype, the* EPHX2* GG genotype group had a higher risk of ischemic stroke, whereas the* EPHX2* GA + AA genotype group did not.

Levels of EETs depend not only on hydrolysis to dihydroxyeicosatrienoic acids (DHETs) by sEH but also on their production by cytochrome P450 enzymes (CYPs). High Hcy level can downregulate CYP2J2 protein expression [34] and upregulate sEH protein expression in vitro and in vivo [24], thus attenuating the protective effect against ischemic stroke by reducing the production of EETs. Interactions between genetic and environmental factors play a substantial role in disease risk [35] and Hcy level may influence the risk of ischemic stroke caused by* EPHX2* polymorphism. In our study, Hcy level was not associated with* EPHX2* rs751141 polymorphism (Supplementary Table  1). However, Hcy level stratification indicated that a low Hcy level has an important genetic effect on rs751141 and therefore on the risk of ischemic stroke. The A allele showed a protective effect in the low Hcy group but not in the medium and high Hcy groups.

Limitations of this study should be mentioned. First, our investigation was performed with 236 T2DM patients with ischemic stroke and 390 control participants. As such, good statistical power was lacking to detect associations, and the sample size of this study was limited when stratified by genotype or Hcy level. Second, this study was conducted in Chinese participants, and whether the results can be generalized to other ethnic groups needs further investigation. Finally, the precise biologic mechanism of the protective effect of the A allele of G860A against ischemic stroke needs further elucidation.

In summary, our findings revealed that the combined effect of* MTHFR* C677T and* EPHX2* G860A genotypes appears to be significantly associated with development of ischemic stroke in the Chinese T2DM group. Pharmacologic inhibition of sEH is being investigated as a novel therapeutic strategy for stroke disease [30]. Therefore, based on our observation, folic acid supplementation [36] and sEH inhibitors may be used selectively to decrease ischemic stroke risk in carriers with high genetic risk.

## Supplementary Material

In terms of the MTHFR C677T genotype, Hcy level is associated with MTHFR C677T polymorphism (*P* = .0497), and TT genotype was associated with higher Hcy level compared with the CC genotype. In terms of the EPHX2 G860A genotype, Hcy level was not associated with EPHX2 rs751141 polymorphism (*P* = .62) (Supplementary Table 1).

## Figures and Tables

**Table 1:** Characteristics of T2DM in ischemic stroke cases and control group.

Variables	Ischemic stroke (*n* = 236)^a^	Control (*n* = 390)^a^	*P*
Age, y	66.0 (60.0, 74.0)	62.0 (55.0, 69.0)	<.001
Sex, male (%)	53.8 (127/236)	61.8 (241/390)	.054
BMI, Kg/m^2^	25.7 (23.5, 28.3)	25.5 (23.5, 28.0)	.960
Duration of diabetes, y	16.0 (10.0, 20.5)	16.0 (12.0, 20.0)	.111
History of hypertension (%)	75.4 (178/236)	58.2 (227/390)	<.001
Current smoking (%)	30.9 (73/236)	25.4 (99/390)	.140
SBP (mmHg)	134.5 (120.0, 146.0)	130.0 (120.0, 143.0)	.014
DBP (mmHg)	80.0 (70.0, 81.0)	80.0 (72.0, 80.0)	.879
A1C (%)	7.7 (6.7, 9.4)	7.9 (6.8, 9.2)	.668
Hcy (*μ*mol/L)	12.6 (10.3, 15.7)	12.2 (9.9, 14.4)	.073
TC (mmol/L)	4.09 (3.35, 4.85)	4.19 (3.55, 4.85)	.306
HDL-C (mmol/L)	1.00 (0.83, 1.22)	1.00 (0.84, 1.24)	.825
LDL-C (mmol/L)	2.25 (1.83, 2.89)	2.39 (1.87, 2.96)	.212
TG (mmol/L)	1.49 (1.07, 2.09)	1.54 (1.08, 2.38)	.263

A1C: hemoglobin A1C; BMI, body mass index; DBP, diastolic blood pressure; Hcy, homocysteine; HDL-C, high density lipoprotein cholesterol; LDL-C, low density lipoprotein cholesterol; SBP, systolic blood pressure; TC, total cholesterol; TG, triglyceride.

^a^Data are shown as median (interquartile range) or %.

**Table 2:** Genotype distribution and allele frequency of *MTHFR* C677T and* EPHX2* G860A in ischemic stroke and control groups.

	Genotype frequencies				Allele frequencies		
*MTHFR*	CC	CT	TT	*P*	C	T	*P*

Ischemic stroke	36	106	94	.020	37.7	62.3	.005
Control	88	183	119		46	54.0	

*EPHX2*	GG	GA	AA	*P*	G	A	*P*

Ischemic stroke	145	79	12	.053	78.2	21.8	.023
Control	201	162	27		72.3	27.7	

**Table 3:** Odds ratios and 95% confidence interval for ischemic stroke under three genetic models.

	Genetic models	Unadjusted		Adjusted^a^		Adjusted^b^	
		OR (95% CI)	*P*	OR (95% CI)	*P*	OR (95% CI)	*P*
*MTHFR*	Additive	1.39 (1.10–1.74)	.005	1.40 (1.11–1.77)	.005	1.42 (1.11–1.81)	.004
	Dominant	1.62 (1.06–2.48)	.027	1.60 (1.04–2.48)	.033	1.62 (1.04–2.52)	.034
	Recessive	1.51 (1.07–2.11)	.017	1.56 (1.10–2.20)	.012	1.60 (1.12–2.29)	.01
	CT versus CC	1.42 (0.90–2.23)	.135	1.38 (0.87–2.20)	.173	1.37 (0.86–2.21)	.189
	TT versus CC	1.93 (1.20–3.10)	.006	1.96 (1.21–3.17)	.006	2.00 (1.22–3.29)	.006
*EPHX2*	Additive	0.75 (0.55–0.95)	.02	0.72 (0.54–0.95)	.02	0.72 (0.55–0.97)	.031
	Dominant	0.67 (0.48–0.93)	.016	0.65 (0.47–0.91)	.012	0.67 (0.48–0.95)	.023
	Recessive	0.72 (0.36–1.45)	.36	0.75 (0.37–1.52)	.418	0.74 (0.36–1.56)	.432
	GA versus GG	0.68 (0.48–0.95)	.025	0.65 (0.46–0.93)	.018	0.68 (0.47–0.97)	.033
	AA versus GG	0.62 (0.30–1.26)	.182	0.63 (0.31–1.30)	.21	0.64 (0.30–1.35)	.239

CI, confidence interval; OR, odds ratio.

^a^Adjusted for age and sex.

^b^Adjusted for age, sex, BMI, history of hypertension, TC, TG, and HDL-C.

**Table 4:** Odds ratios and 95% confidence interval for the combined effect of *MTHFR* C677T and *EPHX2* G860A polymorphism and ischemic stroke.

Genotype		Ischemic stroke	Control	Unadjusted		Adjusted^a^		Adjusted^b^	
*EPHX2*	*MTHFR*	236	390	OR (95% CI)	*P*	OR (95% CI)	*P*	OR (95% CI)	*P*
GG	CC	25	40	2.73 (1.20–6.22)	.017	2.63 (1.13–6.12)	.024	2.38 (1.00–5.65)	.049
GG	CT	70	98	3.12 (1.51–6.43)	.002	3.12 (1.49–6.53)	.003	2.92 (1.37–6.22)	.005
GG	TT	50	63	3.46 (1.63–7.35)	.001	3.41 (1.58–7.35)	.002	3.32 (1.51–7.29)	.003
GA + AA	CC	11	48	1 (Ref)		1 (Ref)		1 (Ref)	
GA + AA	CT	36	85	1.84 (0.86–3.96)	.114	1.67 (0.77–3.62)	.198	1.60 (0.72–3.55)	.246
GA + AA	TT	44	56	3.42 (1.60–7.37)	.002	3.47 (1.59–7.59)	.002	3.37 (1.51–7.50)	.003

^a^Adjusted for age and sex.

^b^Adjusted for age, sex, BMI, history of hypertension, TC, TG, and HDL-C.

**Table 5:** Association of *EPHX2 *G860A with risk of ischemic stroke in different Hcy level groups.

Hcy level (*μ*mol/L)	Genotype	Unadjusted		Adjusted^a^		Adjusted^b^	
		OR (95% CI)	*P*	OR (95% CI)	*P*	OR (95% CI)	*P*
Low: 4.29–10.7 (*n* = 209)	Additive	0.45 (0.27–0.75)	.002	0.45 (0.27–0.76)	.003	0.43 (0.25–0.75)	.003
	Recessive	0.11 (0.01–0.87)	.035	0.10 (0.01–0.81)	.031	0.09 (0.01–0.79)	.030
	Dominant	0.44 (0.24–0.81)	.008	0.45 (0.25–0.83)	.011	0.43 (0.23–0.82)	.011
Medium: 10.74–13.71 (*n* = 208)	Additive	1.16 (0.73–1.84)	.528	1.15 (0.71–1.86)	.579	1.15 (0.69–1.92)	.584
	Recessive	2.33 (0.75–7.22)	.142	2.52 (0.78–8.16)	.124	2.61 (0.74–9.18)	.135
	Dominant	1.01 (0.57–1.80)	.964	0.97 (0.54–1.77)	.931	0.97 (0.51–1.84)	.936
High: 13.73–53.99 (*n* = 209)	Additive	0.69 (0.43–1.11)	.127	0.70 (0.43–1.14)	.152	0.69 (0.42–1.14)	.146
	Recessive	0.86 (0.23–3.14)	.817	0.90 (0.25–3.32)	.879	0.84 (0.21–3.29)	.798
	Dominant	0.62 (0.36–1.38)	.088	0.63 (0.36–1.10)	.102	0.62 (0.35–1.11)	.108

Hcy, homocysteine.

^a^Adjusted for age and sex.

^b^Adjusted for age, sex, BMI, history of hypertension, TC, TG, and HDL-C.

## References

[1] International Diabetes Federation (2016). *IDF Diabetes Atlas*.

[2] Xu Y., Wang L., He J. (2013). Prevalence and control of diabetes in Chinese adults. *JAMA*.

[3] Harmsen P., Lapas G., Rosengren A., Wilhelmsen L. (2006). Long-term risk factors for stroke: twenty-eight years of follow-up of 7457 middle-aged men in Göteborg, Sweden. *Stroke*.

[4] Davis T. M. E., Millns H., Stratton I. M., Holman R. R., Turner R. C., Bassett P. (1999). Risk factors for stroke in type 2 diabetes mellitus: United Kingdom Prospective Diabetes Study (UKPDS) 29. *Archives of Internal Medicine*.

[5] Ma R. C. W., Chan J. C. N. (2013). Type 2 diabetes in East Asians: similarities and differences with populations in Europe and the United States. *Annals of the New York Academy of Sciences*.

[6] Bak S., Gaist D., Sindrup S. H., Skytthe A., Christensen K. (2002). Genetic liability in stroke: a long-term follow-up study of Danish twins. *Stroke*.

[7] Eikelboom J. W., Hankey G. J., Anand S. S., Lofthouse E., Staples N., Baker R. I. (2000). Association between high homocyst(e)ine and ischemic stroke due to large- and small-artery disease but not other etiologic subtypes of ischemic stroke. *Stroke*.

[8] Wang W., Wang Y., Gong F., Zhu W., Fu S. (2013). MTHFR C677T polymorphism and risk of congenital heart defects: evidence from 29 case-control and TDT studies. *PLoS ONE*.

[9] Frosst P., Blom H. J., Milos R. (1995). A candidate genetic risk factor for vascular disease: a common mutation in methylenetetrahydrofolate reductase. *Nature Genetics*.

[10] McIlroy S. P., Dynan K. B., Lawson J. T., Patterson C. C., Passmore A. P. (2002). Moderately elevated plasma homocysteine, methylenetetrahydrofolate reductase genotype, and risk for stroke, vascular dementia, and Alzheimer disease in Northern Ireland. *Stroke*.

[11] They-They T. P., Nadifi S., Rafai M. A., Battas O., Slassi I. (2011). Methylenehydrofolate reductase (C677T) polymorphism and large artery ischemic stroke subtypes. *Acta Neurologica Scandinavica*.

[12] Bentley P., Peck G., Smeeth L., Whittaker J., Sharma P. (2010). Causal relationship of susceptibility genes to ischemic stroke: comparison to ischemic heart disease and biochemical determinants. *PLoS ONE*.

[13] Zhang D., Zhou Y., Han L., Ji H., Li J. (2014). The effect of MTHFR C677T polymorphism on type 2 diabetes mellitus with vascular complications in Chinese Han population: a meta-analysis. *Endocrine Journal*.

[14] Sun J.-Z., Xu Y., Lu H., Zhu Y. (2009). Polymorphism of the methylenetetrahydrofolate reductase gene association with homocysteine and ischemic stroke in type 2 diabetes. *Neurology India*.

[15] Yu Z., Xu F., Huse L. M. (2000). Soluble epoxide hydrolase regulates hydrolysis of vasoactive epoxyeicosatrienoic acids. *Circulation Research*.

[16] Zhang W., Otsuka T., Sugo N. (2008). Soluble epoxide hydrolase gene deletion is protective against experimental cerebral ischemia. *Stroke*.

[17] Imig J. D., Zhao X., Zaharis C. Z. (2005). An orally active epoxide hydrolase inhibitor lowers blood pressure and provides renal protection in salt-sensitive hypertension. *Hypertension*.

[18] Przybyla-Zawislak B. D., Srivastava P. K., Vázquez-Matías J. (2003). Polymorphisms in human soluble epoxide hydrolase. *Molecular Pharmacology*.

[19] Burdon K. P., Lehtinen A. B., Langefeld C. D. (2008). Genetic analysis of the soluble epoxide hydrolase gene, EPHX2, in subclinical cardiovascular disease in the Diabetes Heart Study. *Diabetes and Vascular Disease Research*.

[20] Fornage M., Lee C. R., Doris P. A. (2005). The soluble epoxide hydrolase gene harbors sequence variation associated with susceptibility to and protection from incident ischemic stroke. *Human Molecular Genetics*.

[21] Gschwendtner A., Ripke S., Freilinger T. (2008). Genetic variation in soluble epoxide hydrolase (EPHX2) is associated with an increased risk of ischemic stroke in white Europeans. *Stroke*.

[22] Lee J., Dahl M., Grande P., Tybjærg-Hansen A., Nordestgaard B. G. (2010). Genetically reduced soluble epoxide hydrolase activity and risk of stroke and other cardiovascular disease. *Stroke*.

[23] Zhang L., Ding H., Yan J. (2008). Genetic variation in cytochrome P450 2J2 and soluble epoxide hydrolase and risk of ischemic stroke in a Chinese population. *Pharmacogenetics and Genomics*.

[24] Zhang D., Xie X., Chen Y., Hammock B. D., Kong W., Zhu Y. (2012). Homocysteine upregulates soluble epoxide hydrolase in vascular endothelium in vitro and in vivo. *Circulation Research*.

[25] World Health Organization Department of Noncommunicable Disease Surveillance (1999). *Definition, Diagnosis and Classification of Diabetes Mellitus: Part 1: Diagnosis and Classification of Diabetes Mellitus*.

[26] Lozano R., Naghavi M., Foremanet K. (1990). Global and regional mortality from 235 causes of death for 20 age groups in 1990 and 2010: a systematic analysis for the Global Burden of Disease Study 2010. *The Lancet*.

[27] Sibani S., Christensen B., O'Ferrall E. (2000). Characterization of six novel mutations in the methylenetetrahydrofolate reductase (MTHFR) gene in patients with homocystinuria. *Human Mutation*.

[28] Wilcken B., Bamforth F., Li Z. (2003). Geographical and ethnic variation of the 677C>T allele of 5, 10 methylenetetrahydrofolate reductase (MTHFR): findings from over 7000 newborns from 16 areas world wide. *Journal of Medical Genetics*.

[29] Qin X., Li J., Cui Y. (2012). MTHFR C677T and MTR A2756G polymorphisms and the homocysteine lowering efficacy of different doses of folic acid in hypertensive Chinese adults. *Nutrition Journal*.

[30] Zhang W., Koerner I. P., Noppens R. (2007). Soluble epoxide hydrolase: a novel therapeutic target in stroke. *Journal of Cerebral Blood Flow and Metabolism*.

[31] Zuloaga K. L., Krasnow S. M., Zhu X. (2014). Mechanism of protection by soluble epoxide hydrolase inhibition in type 2 diabetic stroke. *PLoS ONE*.

[32] Koerner I. P., Jacks R., DeBarber A. E. (2007). Polymorphisms in the human soluble epoxide hydrolase gene EPHX2 linked to neuronal survival after ischemic injury. *The Journal of Neuroscience*.

[33] Nelson J. W., Subrahmanyan R. M., Summers S. A., Xiao X., Alkayed N. J. (2013). Soluble epoxide hydrolase dimerization is required for hydrolase activity. *Journal of Biological Chemistry*.

[34] Moshal K. S., Zeldin D. C., Sithu S. D. (2008). Cytochrome P450 (CYP) 2J2 gene transfection attenuates MMP-9 via inhibition of NF-*κβ* in hyperhomocysteinemia. *Journal of Cellular Physiology*.

[35] Rutter M., Moffitt T. E., Caspi A. (2006). Gene-environment interplay and psychopathology: multiple varieties but real effects. *Journal of Child Psychology and Psychiatry and Allied Disciplines*.

[36] Huo Y., Li J., Qin X. (2015). Efficacy of folic acid therapy in primary prevention of stroke among adults with hypertension in China: the CSPPT randomized clinical trial. *The Journal of the American Medical Association*.

