# Supplementary material for: Synergistic Effect of the MTHFR C677T and EPHX2 G860A Polymorphism on the Increased Risk of Ischemic Stroke in Chinese Type 2 Diabetic Patients
Source: J Diabetes Res. 2017 Mar 20;2017:6216205. doi: 10.1155/2017/6216205 (PMC5376931; doi:10.1155/2017/6216205)
Supplement: Supplementary file 1 — In terms of the MTHFR C677T genotype, Hcy level is associated with MTHFR C677T polymorphism (P = .0497), and TT genotype was associated with higher Hcy level compared with the CC genotype. In terms of the EPHX2 G860A genotype, Hcy level was not associated with EPHX2 rs751141 polymorphism (P = .62) (Supplementary Table 1). [file 6216205.f1.pdf]

Supplementary table 1. Effect of rs751141and rs1801133 polymorphisms on homocysteine levels

**Genotype**	**Hcy level (μmol/L)**	***P*[a]**	**β (SE)[a]**
*MTHFR*rs751141(n=626)			
CC	11.77 (9.87, 14.78)	.0497	0.57 (0.29)
CT	12.07 (9.88, 14.44)		
TT	12.80 (10.53, 15.80)		
*EPHX2*rs1801133(n=626)			
GG	12.20 (9.78, 14.92)	.62	0.17 (0.34)
GA	12.56 (10.49, 14.94)		
AA	11.38 (9.34, 14.67)		

[a]Adjusted for age, sex.
